# A Position Statement on Endovascular Models and Effectiveness Metrics for Mechanical Thrombectomy Navigation, on Behalf of the Stakeholder Taskforce for Artificial Intelligence–Assisted Robotic Thrombectomy (START)

**DOI:** 10.1161/JAHA.125.044931

**Published:** 2026-03-28

**Authors:** Harry Robertshaw, Anna Barnes, Phil Blakelock, Raphael Blanc, Robert Crossley, Rebecca Fahrig, Ameer E. Hassan, Benjamin Jackson, Lennart Karstensen, Neelam Kaur, Markus Kowarschik, Jeremy Lynch, Franziska Mathis‐Ullrich, Dwight Meglan, Vitor Mendes Pereira, Mouloud Ourak, Matteo Pantano, S. M. Hadi Sadati, Alice Taylor‐Gee, Tom Vercauteren, Phil White, Alejandro Granados, Thomas C. Booth, START, Frédéric Clarençon, Adnan Siddiqui, David Bell, Nikola Fischer, Kawal Rhodes, Christos Bergeles

**Affiliations:** ^1^ School of Biomedical Engineering and Imaging Sciences Kings College London London UK; ^2^ King’s Technology Evaluation Centre, School of Biomedical Engineering and Imaging Sciences Kings College London London UK; ^3^ Patient and Public Involvement Kings College London London UK; ^4^ Department of Interventional Neuroradiology Rothschild Foundation Hospital Paris France; ^5^ Department of Interventional Neuroradiology North Bristol NHS Trust Bristol UK; ^6^ Siemens Healthineers AG Erlangen Germany; ^7^ Pattern Recognition Lab, Department of Computer Science Friedrich‐Alexander University Erlangen‐Nurnberg Erlangen Germany; ^8^ Department of Neurology University of Texas Rio Grande Valley Edinburg TX; ^9^ Society of Vascular and Interventional Neurology Minneapolis MN; ^10^ Department Artificial Intelligence in Biomedical Engineering Friedrich‐Alexander University Erlangen‐Nurnberg Erlangen Germany; ^11^ Stroke Association London UK; ^12^ European Society of Minimally Invasive Neurological Therapy Zurich Switzerland; ^13^ Department of Neuroradiology Kings College Hospital London UK; ^14^ Department of Neuroradiology Queens Hospital Romford London UK; ^15^ Exemplar Devices LLC Beavercreek OH; ^16^ Divisions of Therapeutic Neuroradiology and Neurosurgery St. Michael’s Hospital Toronto ON Canada; ^17^ Department of Mechanical Engineering KU Leuven University Leuven Belgium; ^18^ School of Engineering and Materials Science Queen Mary University of London London UK; ^19^ Translational and Clinical Research Institute Newcastle University Newcastle UK; ^20^ Newcastle upon Tyne Hospitals NHS Foundation Trust Newcastle UK; ^21^ United Kingdom Neurointerventional Group London UK

**Keywords:** artificial intelligence, endovascular intervention, machine learning, mechanical thrombectomy, robotics, stroke, Ischemic Stroke, Cerebrovascular Disease/Stroke, Cerebrovascular Procedures, Revascularization, Machine Learning

## Abstract

Although we are making progress in overcoming infectious diseases and cancer, one of the major medical challenges of the mid‐21st century will be the increasing prevalence of stroke. Occlusions in large vessels are especially debilitating, yet effective treatment—needed within hours to achieve best outcomes—remains limited because of geographic accessibility. One solution for improving timely access to mechanical thrombectomy in geographically diverse populations is the widespread deployment of robotic surgical systems. Artificial intelligence assistance may enable the safe and effective upskilling of operators in this emerging therapeutic delivery approach. Our aim was to establish consensus frameworks for developing and validating artificial intelligence–assisted robots for thrombectomy. Objectives included standardizing effectiveness metrics and defining reference testbeds across in silico, in vitro, ex vivo, and in vivo environments. To achieve this, we convened experts in neurointervention, robotics, data science, health economics, policy, statistics, and patient advocacy. Consensus was built through an incubator day, a Delphi process, and a final position statement. We identified that the 4 essential testbed environments each had distinct validation roles. Realism requirements vary: simpler testbeds should include realistic vessel anatomy compatible with guidewire and catheter use, whereas standard testbeds should incorporate deformable vessels. More advanced testbeds should include blood flow, pulsatility, and disease features, such as atheromatous plaques. There are 2 macroclasses of effectiveness metrics: one for in silico, in vitro, and ex vivo stages focusing on technical navigation (eg, path‐following error), and another for in vivo stages, focused on clinical outcomes (eg, modified treatment in cerebral infarction scores). Patient safety is central, and not a barrier, to this technology's development. One requisite patient safety task needed now is to correlate in vitro measurements to in vivo complications.

**Nonstandard Abbreviations and Acronyms**
eTICI: extended Treatment in Cerebral Infarction
ML: machine learning
MT: mechanical thrombectomy
mTICI: modified Treatment in Cerebral Infarction
START: Stakeholder Taskforce for Artificial Intelligence–Assisted Robotic Thrombectomy
TRL: technology readiness level

The annual number of strokes and stroke‐related deaths increased by 70% and 43%, respectively, from 1990 to 2019, making stroke the second‐leading cause of death and the third highest contributor to the burden of disease worldwide.^1^, ^2^ Mechanical thrombectomy (MT) has emerged as a standard treatment for acute ischemic stroke resulting from large‐vessel occlusion, providing improved functional outcomes when compared with medical treatment alone.^3^, ^4^, ^5^, ^6^

In cases of ischemic stroke, the timing of intervention from symptom onset plays a critical role in the effectiveness of MT. Notably, the efficacy of MT diminishes significantly after 7.3 hours from stroke onset for nonstratified patients.^7^ Despite recent evidence suggesting a growing proportion of patients with stroke qualifying for MT,^5^ and despite the potential benefits of MT, there is a significant gap in treatment accessibility seen within all countries.^8^, ^9^, ^10^ Even in high‐income countries a significant gap exists. In the United Kingdom, for example, only 3.1% of stroke admissions received MT in 2023, despite 10% being eligible.^8^, ^9^ Patients arriving from remote stroke centers are less likely to receive MT if the door‐to‐door time is >3 hours, which occurs in 45% of admissions.^11^ Similar findings have been seen in the United States, where the probability of undergoing MT decreases by 1% for each additional minute of transfer time over an ideal transfer time of 1 hour.^12^

Challenges associated with MT include occasional complications, such as vessel perforations (1%), procedure‐related vessel dissections (2%), and distal embolization of thrombus (9%).^13^ Beyond patient safety, operators and their teams face the risk of potentially high cumulative doses of X‐ray radiation from angiography, which pose risks of cancer and cataracts.^14^ Although current radiation protection practices help minimize exposure, some measures, such as wearing heavy protective equipment, can lead to orthopedic complications.^15^, ^16^ One proposed solution that mitigates these challenges while ensuring timely access to MT for diverse populations involves the deployment of cost‐effective robotic surgical systems, which could be strategically positioned in hospitals nationwide and then operated remotely from a central hub by experts, operated with artificial intelligence (AI) assistance by competent but nonexpert operators, or even autonomously.^17^ This would greatly increase access to treatment, where currently 31.2% of the US population have no access to an interventional radiologist within their county.^18^ Robotic interventions executed by trained operators offer several advantages, including the potential elimination of operator tremors, reduced radiation exposure, and enhanced procedural precision, thereby minimizing complications.^19^ Robotically actuated systems have demonstrated successful outcomes in neuroendovascular interventions.^20^, ^21^ However, robotically actuated systems that use a controller‐operator structure may lead to high cognitive workloads with the potential for human error,^22^ although this has not yet been proven for endovascular robotics.

Integrating AI techniques with robotic actuation systems has emerged as a promising approach to reduce risk and increase clinical acceptance of robotic surgical systems. Machine learning (ML) has seen rapid advancement, offering potential solutions for assisted navigation of guidewires and catheters during MT.^23^ ML algorithms may provide assistance or autonomy in navigation, mitigating the challenges associated with full manual control, including human factors, such as fatigue and loss of focus, potentially enhancing procedural safety and efficiency.^24^ Additionally, autonomous or assisted navigation might upskill local generalist operators rather than relying on teleoperation by experts from central hubs. Therefore, AI assistance democratizes the ability of all operators, while minimizing clinical differences in operator technique and potentially judgment too.

Although several studies have explored the integration of ML in automating catheter and guidewire manipulation for endovascular interventions, a recent systematic review highlighted the lack of high‐level evidence to demonstrate that AI‐based autonomous navigation of catheters and guidewires in any endovascular intervention is noninferior or superior to manual procedures.^25^, ^26^ The review found that the field has not surpassed an experimental proof‐of‐concept stage with a technology readiness level (TRL) of 3,^27^ which corresponds to active research and development efforts that involve both analytical studies to place the technology in context and laboratory‐based experiments to validate the predictions made during the conceptual phase (TRL 2).^27^ Furthermore, the review found that there are no standardized in silico, in vitro, ex vivo, or in vivo experimental reference standard testbeds, nor are there standardized effectiveness metrics, meaning that comparison of studies quantitatively is of limited value.

To increase the TRL of the field, and move toward demonstrating any benefits of ML for the autonomous navigation of MT equipment, it is vital to establish reference and reporting standards for robotic MT. Such frameworks will also be suitable for a wide range of MT development regardless of whether robots or ML is used.

The purpose of this position statement was to establish, through consensus, reference and reporting standard frameworks to be used for the development and validation of robotic MT, both with and without AI. Objectives were to standardize effectiveness metrics (including determining standardized units of measurement) and reference standard testbeds for in silico, in vitro, ex vivo, and in vivo environments.

## METHODS

### Incubator Meeting

A multidisciplinary group comprising health care professionals, academia and industry experts, and patient advocacy representatives convened for an incubator day on AI‐informed robotics for MT in ischemic stroke. The first meeting took place in London, UK, in April 2024, with the option to attend virtually to allow for international attendance. There were 21 expert attendees, invited based on their expertise in neuroendovascular procedures, robotics, data science, health economics, health care policy, statistics, and patient advocacy.

Current endovascular testbed reference standards and effectiveness metrics, for in silico, in vitro, ex vivo, and in vivo environments, were reviewed and discussed among the group, where it was concluded that standardization among these areas is needed to improve AI research outcomes.^25^ Additionally, current MT clinical effectiveness metrics were reviewed,^4^, ^7^ and consideration was given to what metrics could be used in early TRL research to give the highest likelihood of improved clinical outcomes at later translational stages.^28^

On the basis of this meeting, a consensus was reached that a Delphi study should be conducted in the first instance to establish standards for neuroendovascular testbeds and effectiveness metrics across in silico, in vitro, ex vivo, and in vivo environments for autonomous robotics in MT. Following this, it was agreed that the consensus report of a Delphi study^29^ would form the basis of the position statement.^30^ The position statement is particularly well suited to promoting discussion on emerging topics and identifying evidence gaps, as well as highlighting current strengths and limitations in the field. Together, this would provide the foundation for producing a future multistakeholder guideline^30^ that builds on the current position statement.

### Delphi Method

A Delphi consensus is commonly used to gain agreement across a panel of experts, and was used here to provide anonymity of the panel, while ensuring that each participant had an equal possibility to provide and change their opinion during the course of the process.^31^, ^32^ All members of the group who attended the incubator meeting were invited to be Delphi panelists.

**Figure 1:**
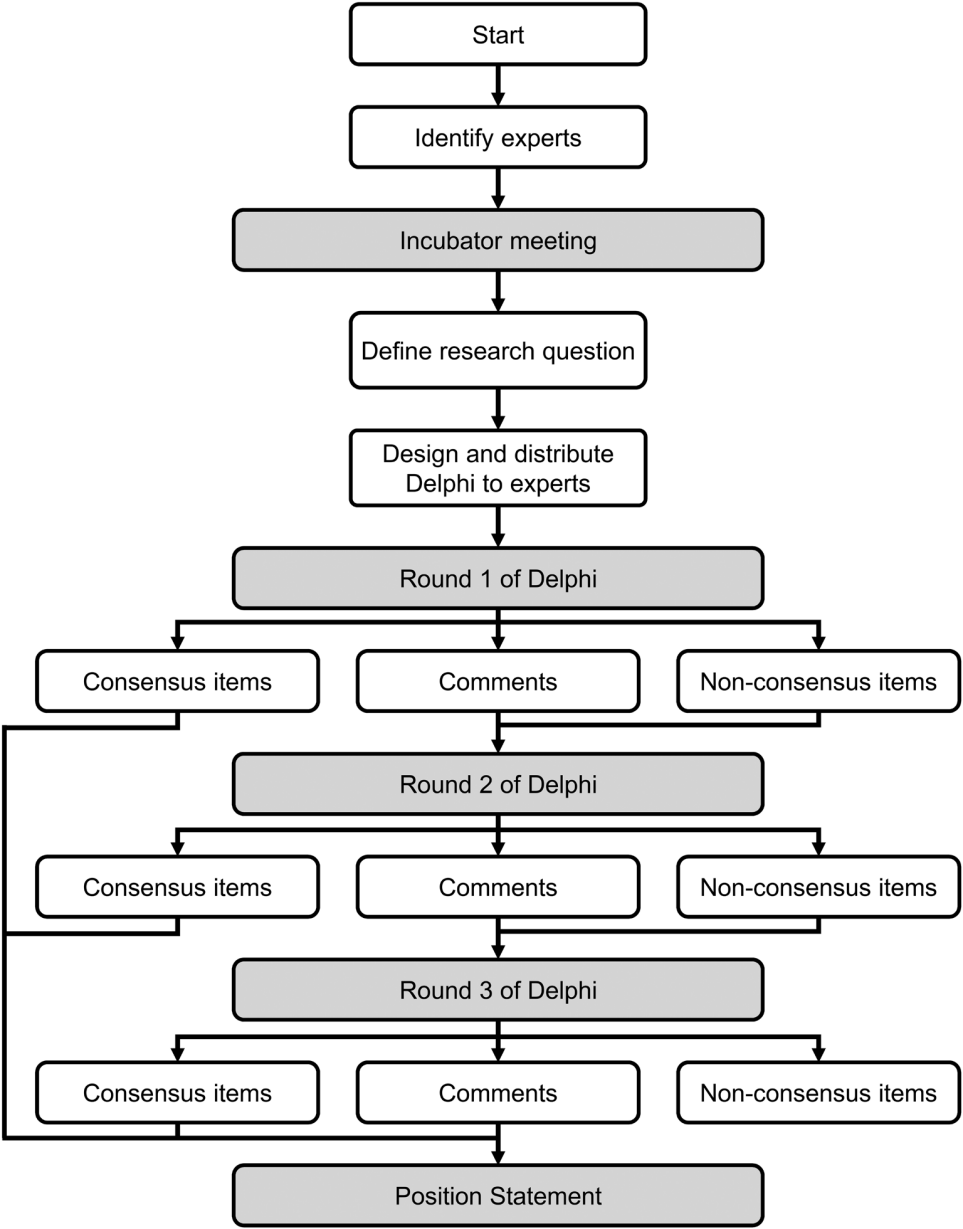
Flowchart for methods followed in this study, including incubator meeting, 3 rounds of Delphi, and final position statement.

The Delphi exercise consisted of 3 rounds.^33^ The process followed is shown in Figure 1. The first round was sent by email to all panelists in May 2024. For all rounds, a reminder was sent 2 weeks later to individuals who did not respond to the initial invite. The second round was distributed in July 2024. Any questions that reached 80% consensus in the previous round were considered as finalized and were removed from the questionnaire. The remaining questions, which had <80% consensus, were included in the questionnaire; and contributors were informed of the current percentage of agreement from the previous round. New options were also added to the questionnaire on the basis of panelist feedback through free‐text comments from the previous round. The same process was followed for round 3, which was sent out in September 2024.

The survey was split into 4 sections. In section 1, panelists were asked to provide baseline information about their experience with MT. In section 2, participants evaluated various aspects of robotic MT, including benefits, risks, and barriers to standardization across different phases of AI‐integrated MT. Questions covered various configurations of robotic MT, including systems both with and without AI. Panelists were asked to consider each factor independently, without assuming other factors were present, and were encouraged to propose additional benefits, risks, or factors requiring standardization.

**Figure 2:**
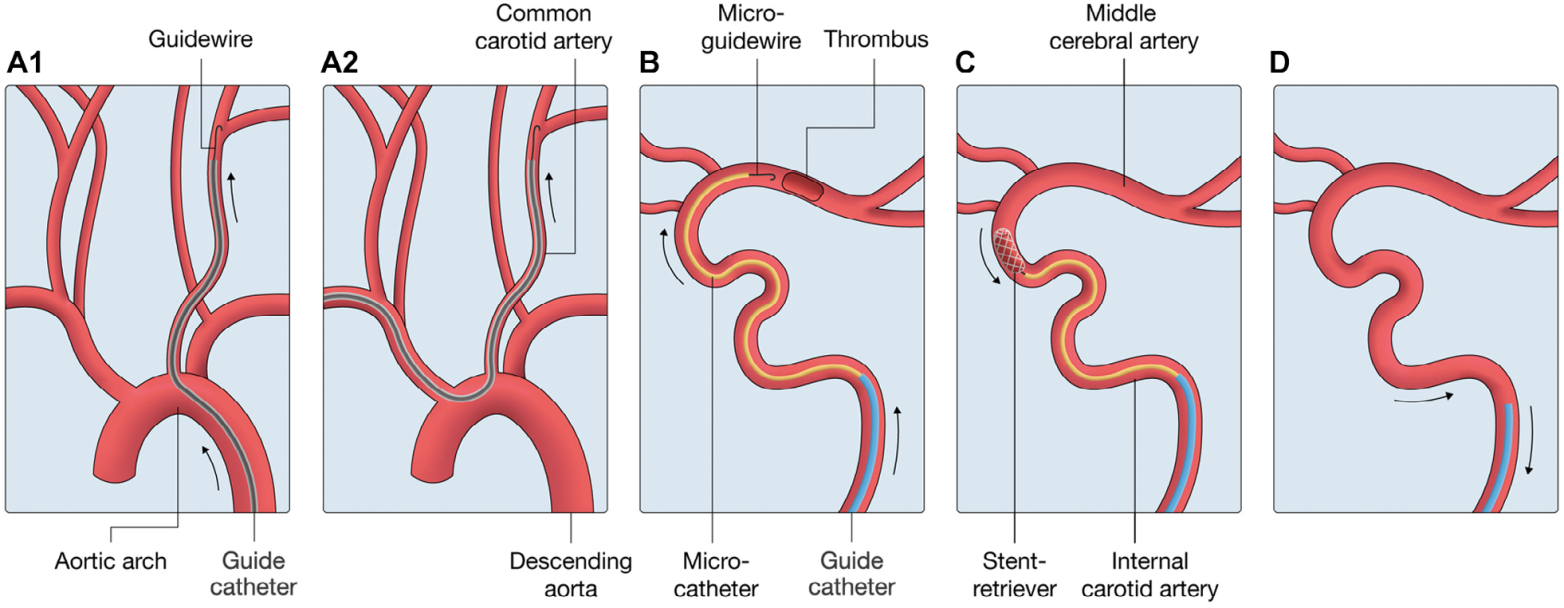
Defined phases of MT intervention: (A1) primary access (femoral artery), (A2) primary access (radial artery), (B) secondary access, (C) treatment (stent shown here, but could also be an aspiration catheter), and (D) removal of navigation equipment (and access closure). The “navigation phases” are considered to be (A) primary and (B) secondary access.

Section 3 focused on neuroendovascular testbed reference standards (also referred to as benchmarks). Panelists separately evaluated different developmental stages of the innovation lifecycle, which consisted of in silico, in vitro, ex vivo, (focusing on human cadavers), and in vivo (testbeds, by definition, must be nonhuman) environments. Within each developmental stage, defined phases^34^ of anterior circulation MT (Figure 2) were considered:

A1) Primary access, using guidewire and guide catheter: femoral artery to common (or internal) carotid artery.

A2) Primary access, using guidewire and guide catheter: radial artery to common (or internal) carotid artery.
B) Secondary access, refers to a phase following primary access, microcatheter, and microguidewire/or aspiration catheter and wire: internal carotid artery to cerebral artery.
C) Treatment, using stent retriever or aspiration catheter.
D) Removal of navigation equipment, and access closure.

The panelists rated the importance of specific testbed features within each environment type, such as realistic vessel anatomy, deformable vessels, simulated blood flow, pulsatility, respiration, use of both catheter and guidewire, and representation of the diseased vessel (eg, atherosclerotic plaques).

Section 4 of the survey assessed the utility of various effectiveness metrics in each type of environment to evaluate robotic MT procedures. The initially proposed MT navigation effectiveness metrics for in silico, in vitro, ex vivo, and in vivo environments were derived from neuroendovascular simulation research^28^ as well as a recent systemic review that captured all metrics published so far in the field of endovascular AI assistance.^25^ Panelists rated the following metrics for their utility as standards to be considered predominantly for the 2 “navigation phases” (primary [A] and secondary [B] access):

- Success rate: how many times can the robot reach the target in a given number of evaluations.
- Number of procedural phases: number of MT procedural phases completed in a navigation attempt (Figure 2), as defined by Crossley et al.^28^
- Number of failures: number of failures made during a navigation attempt (eg, wrong branch catheterization).
- Number of handling errors: number of MT handling errors made in a navigation attempt, as defined by Crossley et al.^28^
- Procedure time.
- Path length: length of path taken by the device tip from insertion point to target.
- Path following error: difference between the catheter tip path to the vessel centerline.
- Instrument tip speed.
- Instrument tip acceleration.
- Instrument tip contact forces: mean and maximum contact forces applied to the tip of the instrument during a navigation attempt.
- Instrument base contact forces: mean and maximum contact forces applied to the base of the instrument during a navigation attempt.
- Vessel wall contact forces: mean and maximum contact forces applied to the vessel walls during a navigation attempt.
- Fluoroscopy time.
- Contrast agent volume.
- Number of guidewire tip touches on vessel walls.

Although we considered what an appropriate nonhuman in vivo testbed might consist of during the Delphi process, we also noted the paucity of nonhuman in vivo testbed and effectiveness metrics described in the literature for MT development, and the well‐defined clinical assessment metrics published in numerous MT trials. These clinical assessment metrics covered criteria, such as mTICI (modified Treatment in Cerebral Infarction) scores,^35^ eTICI (extended Treatment in Cerebral Infarction) scores,^36^ first‐pass success rates, vessel perforation, dissection, hemorrhage, distal embolization, and procedural failure.^13^ These MT clinical effectiveness metrics are the most important metrics for translation as they have been used to derive the level 1 evidence^26^ for MT.^4^, ^7^ The metrics relate to safety and efficacy following an entire MT procedure,^4^, ^7^ and not navigation steps. Therefore, in our Delphi process, in vivo effectiveness metric questions focused on clinical effectiveness and not navigation. In contrast, navigation research may be more suitable for in silico, in vitro, and ex vivo environments, and here effectiveness metric questions focused on navigation during the Delphi process.

Answers to questions from all 4 sections were either binary (yes or no) or used a 5‐point Likert scale (eg, strongly agree, agree, neither agree nor disagree, disagree, or strongly disagree). All questions had an additional “unsure” option and space for free‐text comments for panelists to make suggestions for a future round or to provide comments. Those who selected unsure for an answer (reflecting lack of expertise in that particular domain) were not included in the consensus calculation for that particular question.

Comparative statistics included the χ^2^ test. Significance was set at *P*=0.05. Statistical analyses were conducted using R version 4.3 (R Foundation for Statistical Computing, Vienna, Austria).

## RESULTS

### Delphi Panel

Of the 21 experts who attended the incubator meeting, 20 opted to participate in the Delphi (1 charity representative attendee considered the specialist Delphi exercise beyond his/her technical capability; but the charity has contributed to the final position statement). Two more panelists were added to the Delphi consensus based on recommendations following the incubator meeting. Of the 22 experts who took part in the Delphi study, 22 (100%) completed rounds 1, 2, and 3. Most panelists were academic researchers (64% [14/22]), followed by clinicians (32% [7/22]), data scientists/engineers (23% [5/22]), industry representatives (14% [3/22]), patient representatives (9% [2/22]), and statisticians (5% [1/22]) (panelists were able to identify as being an expert from >1 background). European panelists made up 82% (18/22) of participants, whereas 18% (4/22) were from North America. Most panelists had observed or performed MT procedures (59% [13/22]).

### Robotic MT and AI

Almost all respondents recognized the potential benefits of robotic MT, with 95% (21/22) endorsing robotic MT without AI, and 100% (22/22) endorsing robotic MT with AI assistance in the broadest sense. Notably, AI‐assisted robotic MT for navigation was endorsed as a beneficial tool for 2 broad use cases: for use by neurointerventional experts in a teleoperated capacity (91% [20/22]), and for use by general interventionalists (ie, those performing endovascular procedures but not endovascular procedures related to ischemic or hemorrhagic stroke) who would be upskilled by the technology (95% [21/22]). Fully autonomous robotic MT elicited support of 86% (19/22) by the end of the Delphi exercise. Participants unanimously agreed that it was important to standardize neuroendovascular testbeds and effectiveness metrics for in silico, in vitro, ex vivo, and in vivo environments.

**Figure 3:**
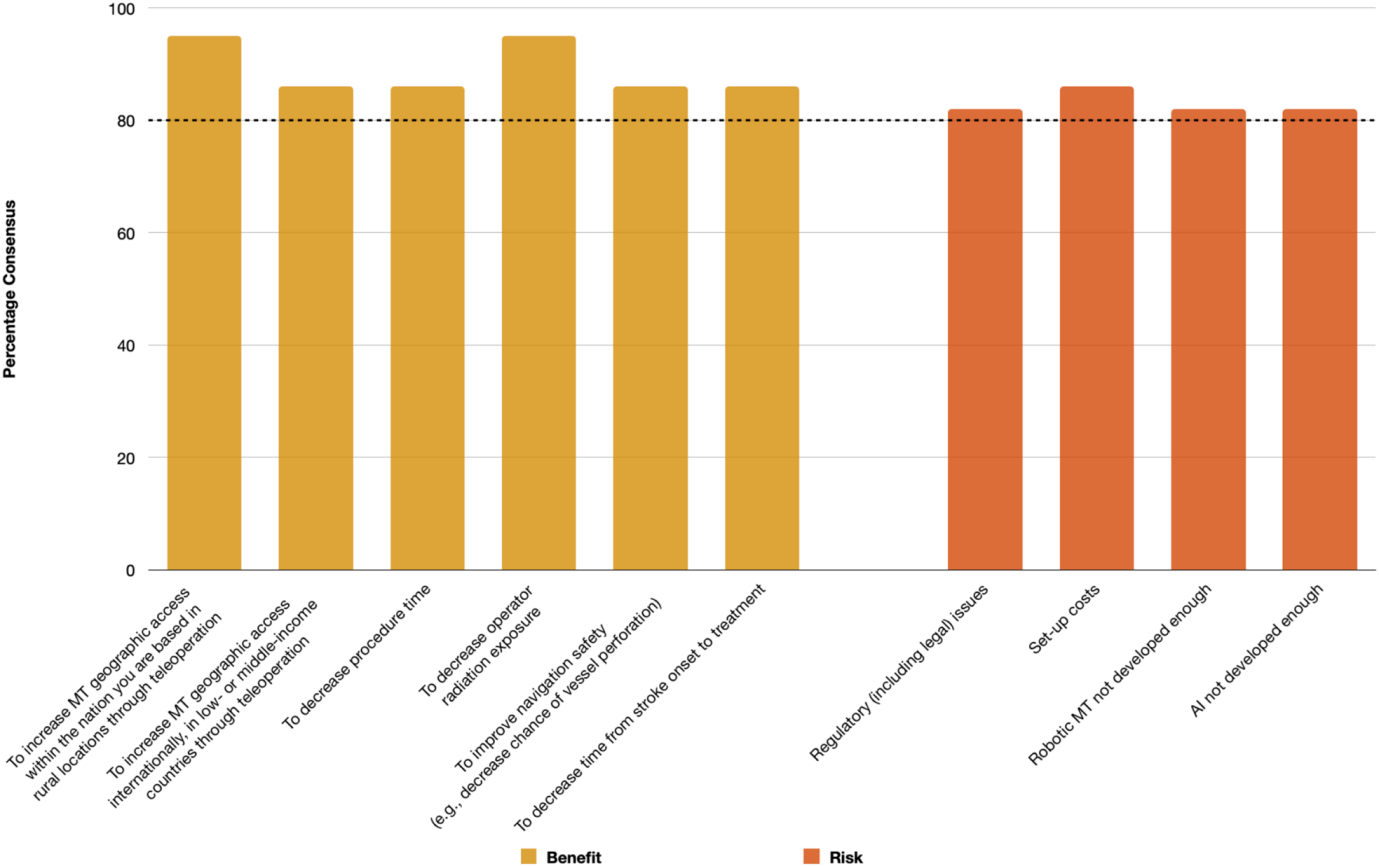
Consensually agreed benefits and risks of robotic MT, both with and without AI (across all developmental stages of robotic MT from in silico to in vivo). Percentage agreement for each benefit and risk was taken from the round where consensus was first reached. Benefits and risks of robotic MT that did not reach consensus in any round can be found in Table S1. AI indicates artificial intelligence; and MT, mechanical thrombectomy.

Figure 3 shows the consensually agreed potential benefits and risks of robotic MT, both with and without AI (across all types of robotic MT); Table S1 shows the benefits and risks that did not reach consensus. One notable benefit endorsed during the Delphi exercise was the ability of robotic MT to increase access in rural locations via teleoperation, which was supported by 95% (21/22) of participants. Additional perceived benefits included reducing operator radiation exposure (95% [21/22]), decreasing procedure time (86% [19/22]), decreasing time from stroke onset to treatment (86% [19/22]), enhancing navigation safety (86% [19/22]), and international access for low‐ and middle‐income countries (86% [19/22]). However, a reduction in patient radiation and contrast exposure was not perceived to be a potential benefit from using robotic MT (with or without AI). Legal and regulatory concerns (82% [18/22]), insufficient development of robotic MT (82% [18/22]), set‐up costs (86% [19/22]), and insufficient development of AI (82% [18/22]) were considered to be risks associated with AI‐based MT navigation not succeeding. Ethical concerns, patient safety, and societal concerns were not considered to be risks per se to AI‐based MT navigation succeeding. (Note: in contrast, patient safety was considered the most important consideration in the position statement overall and is discussed below. Similarly, ethical concerns related to implementation warranted a future position statement.)

### Neuroendovascular Testbeds

**Figure 4:**
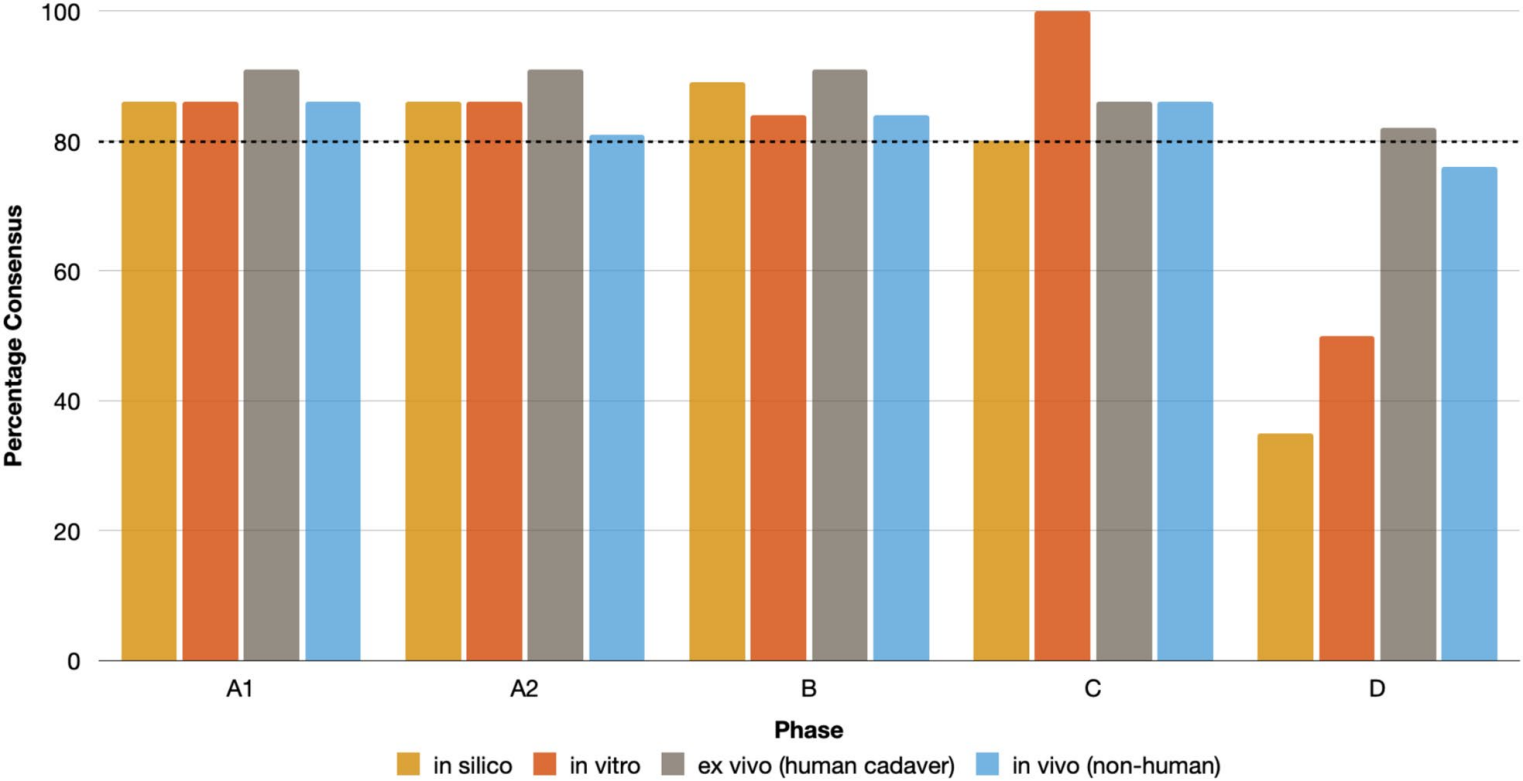
Consensus of which MT phases are effective for the development of robotic MT both with and without artificial intelligence (across all developmental stages of robotic MT from in silico to in vivo), for testbeds from each developmental stage. Percentage agreement for each phase was taken from the round where consensus was first reached. MT indicates mechanical thrombectomy.

The panel considered whether testbeds at each developmental stage (in silico through to in vivo) were appropriate environments to benchmark robotic MT, both with and without AI (Figure 4). The MT phases (A1, A2, B, C, and D) were considered at each developmental stage. Effective design of robotic MT during phases A1, A2, B, and C was considered achievable using all 4 developmental stage testbeds. It was noted that there was no relationship between these phases and developmental stage (A1 [*χ* ^2^ {1, *N*=86}=0.37, *P*=0.95], A2 [*χ* ^2^ {1, *N*=87}=0.90, *P*=0.83], B [*χ* ^2^ {1, *N*=79}=0.67, *P*=0.88], or C [*χ* ^2^ {1, *N*=86}=2.30, *P*=0.51]). In contrast, developing robotic MT to achieve phase D (removal of instruments and access closure) was only considered effective in ex vivo (cadaver) environments (82% [18/22]).

Table 1 lists the experimental factors considered important for testbeds from each developmental stage (both with and without AI). Experimental factors that did not reach consensus in any round can be found in Table S2. Realistic anatomy (including diseased vessels), vessel deformability, and combined use of catheters and guidewires together were considered important factors to be incorporated into all 4 developmental stage testbeds. Simulated blood flow was also supported in testbeds, whereas simulated respiration was not (note that blood flow, pulsatility, and respiration would not need to be simulated in vivo).

**Table 1:** Consensus of Which Experimental Factors Were Considered Important for Each Developmental Stage Testbed (Both With and Without AI)

Experimental factor	In silico	In vitro	Ex vivo	In vivo (nonhuman)
Realistic vessel anatomy/human‐like vessel anatomy	84 (16/19)	95 (18/19)	84 (16/19)	95 (21/22)
Deformable vessels	89 (17/19)	84 (16/19)	84 (16/19)	84 (16/19)
Simulated blood flow	82 (18/22)	84 (16/19)	86 (18/21)	N/A
Simulated pulsatility	86 (18/21)	95 (19/20)	—	N/A
Use of both catheter and guidewire for navigation as opposed to single item	95 (18/19)	95 (18/19)	89 (17/19)	89 (17/19)
Diseased vessel containing atherosclerotic plaques	90 (19/21)	86 (19/22)	80 (16/20)	90 (18/20)

Data are given as percentage (number/total). Percentage agreement for each factor was taken from the round where consensus was first reached. Note that blood flow and pulsatility would not need to be simulated in vivo. Experimental factors that did not reach consensus in any round can be found in Table S2. AI indicates artificial intelligence; N/A, not applicable.

### Effectiveness Metrics

The effectiveness measures selected by consensus for each developmental stage are shown in Table 2.^28^ Measures that did not reach consensus in any round can be found in Table S3.

**Table 2:** Consensus on Important Effectiveness Measures for Each Developmental Stage (With and Without AI), as Well as During in Vivo Clinical‐Like Assessment

Effectiveness measure	In silico	In vitro	Ex vivo	In vivo
Success rate	100 (20/20)	100 (19/19)	100 (19/19)	N/A
No. of phases or steps^28^	84 (16/19)	90 (19/21)	84 (16/19)	N/A
No. of failures (eg, wrong branch catheterization)	95 (19/20)	89 (17/19)	89 (17/19)	N/A
No. of handling errors made^28^	84 (16/19)	89 (17/19)	95 (18/19)	N/A
Procedure time	85 (17/20)	89 (17/19)	86 (19/22)	N/A
Path following error	81 (17/21)	81 (17/21)	—	N/A
Path length	—	81 (17/21)	—	N/A
Instrument tip speed	—	80 (16/20)	—	N/A
Contact forces at instrument tip	89 (16/18)	82 (14/17)	86 (18/21)	N/A
Contact forces at instrument base	83 (15/18)	82 (18/22)	90 (18/20)	N/A
Contact forces on vessel walls	84 (16/19)	86 (19/22)	86 (18/21)	N/A
Fluoroscopy time	90 (19/21)	—	80 (16/20)	N/A
mTICI raw score	N/A	N/A	N/A	92 (11/12)
eTICI raw score	N/A	N/A	N/A	83 (10/12)
“Successful recanalization” first pass (mTICI *≥*2b, after 1 pass)	N/A	N/A	N/A	91 (10/11)
“Complete recanalization” first pass (mTICI *≥*2c, after 1 pass)	N/A	N/A	N/A	91 (10/11)
“Successful recanalization” (mTICI *≥*2b, after *≥*1 pass)	N/A	N/A	N/A	82 (9/11)
“Complete recanalization” (mTICI *≥*2c, after *≥*1 pass)	N/A	N/A	N/A	91 (10/11)
Vessel perforation	N/A	N/A	N/A	100 (18/18)
Vessel dissection	N/A	N/A	N/A	94 (16/17)
Intracranial hemorrhage	N/A	N/A	N/A	94 (16/17)
Distal embolization	N/A	N/A	N/A	100 (16/16)
Procedural failure	N/A	N/A	N/A	94 (17/18)

Data are given as percentage (number/total). Percentage agreement for each factor was taken from the round where consensus was first reached. Measures that did not reach consensus in any round can be found in Table S3.

AI indicates artificial intelligence; eTICI, extended Treatment in Cerebral Infarction; mTICI, modified Treatment in Cerebral Infarction; and N/A, not applicable.

Four conclusions can be drawn from the Delphi exercise. First, we propose that there are multiple effectiveness metrics that should be measured during in silico, in vitro, and ex vivo developmental stages for navigation research. Navigation metrics are related to process (success rate, number of phases or steps, number of failures, and number of handling errors made), duration (procedure time), and contact forces (at instrument tip, at instrument base, and on vessel walls). Similarly, we propose that there are multiple clinical effectiveness metrics that should be measured during in vivo research and are suited for measurement of the entire MT procedure; these can be applied to nonhuman as well as clinical (human) research.

Second, of the effectiveness metrics considered to be essential for navigation research at all developmental stages, success rate, defined as the number of successful attempts in a given number of evaluations, was the most important metric, reaching 100% (20/20) at every stage.

Third, 4 effectiveness metrics were not considered to be essential at all developmental stages (path following error, path length, instrument tip speed, and fluoroscopy time) by the Delphi panel. However, they were considered important at certain developmental stages (eg, fluoroscopy time was considered important during in silico and ex vivo testing, but not in vitro testing). Additionally, several effectiveness metrics were not considered to be essential across any developmental stage. These included volume of the contrast agent used, instrument tip acceleration, and number of contacts between the guidewire tip and vessel wall.

Fourth, all in vivo clinical assessment metrics assessed, including efficacy TICI (Treatment in Cerebral Infarction) scores, complications, and procedural failure, reached consensus.

### Consensus Summary

On the basis of the consensus agreements reached in the Delphi exercise, we summarized and organized the information by developmental stage for user reference (Table 3).

**Table 3:** Combined Consensus Recommendations for Testbeds and Effectiveness Measures Across Developmental Stages

Item	In silico	In vitro	Ex vivo	In vivo
MT navigation phases (in vivo refers to nonhuman)				
Phase A1	✓	✓	✓	✓
Phase A2	✓	✓	✓	✓
Phase B	✓	✓	✓	✓
Phase C	✓	✓	✓	✓
Phase D	—	—	✓	—
Experimental considerations (in vivo refers to nonhuman)				
Realistic vessel anatomy	✓	✓	✓	✓
Deformable vessels	✓	✓	✓	✓
Simulated blood flow	✓	✓	✓	N/A
Simulated pulsatility	✓	✓	—	N/A
Use of catheter and guidewire	✓	✓	✓	✓
Diseased vessel (plaques)	✓	✓	✓	✓
Effectiveness measures				
Success rate, %	✓	✓	✓	N/A
No. of phases or steps	✓	✓	✓	N/A
No. of failures	✓	✓	✓	N/A
Handling errors made	✓	✓	✓	N/A
Procedure time, s	✓	✓	✓	N/A
Path following error, %	✓	✓	—	N/A
Path length, m	—	✓	—	N/A
Instrument tip speed, m/s	—	✓	—	N/A
Contact forces at tip, N	✓	✓	✓	N/A
Contact forces at base, N	✓	✓	✓	N/A
Contact forces on vessel walls, N	✓	✓	✓	N/A
Fluoroscopy time, s	✓	—	✓	N/A
Effectiveness measures (focus on clinical assessment of entire MT procedure; human and nonhuman)				
mTICI raw score	N/A	N/A	N/A	✓
eTICI raw score	N/A	N/A	N/A	✓
“Successful recanalization” first pass (mTICI *≥*2b, after 1 pass)	N/A	N/A	N/A	✓
“Complete recanalization” first pass (mTICI *≥*2c, after 1 pass)	N/A	N/A	N/A	✓
“Successful recanalization” (mTICI *≥*2b, after *≥*1 pass)	N/A	N/A	N/A	✓
“Complete recanalization” (mTICI *≥*2c, after *≥*1 pass)	N/A	N/A	N/A	✓
Vessel perforation	N/A	N/A	N/A	✓
Vessel dissection	N/A	N/A	N/A	✓
Intracranial hemorrhage	N/A	N/A	N/A	✓
Distal embolization	N/A	N/A	N/A	✓
Procedural failure	N/A	N/A	N/A	✓

Units of measure are also given. eTICI indicates extended Treatment in Cerebral Infarction; MT, mechanical thrombectomy; mTICI, modified Treatment in Cerebral Infarction; and N/A, not applicable.

## DISCUSSION

The Delphi consensus recommendations were synthesized by the panel and informed the following positions. We first considered endovascular testbeds and effectiveness metrics in depth in keeping with the position statement objectives. In addition, panelists summarized additional perspectives related to AI‐assisted robotic MT, to highlight key points and flag them for future analyses by Stakeholder Taskforce for AI‐Assisted Robotic Thrombectomy (START) initiatives.

### Endovascular Testbeds

**Figure 5:**
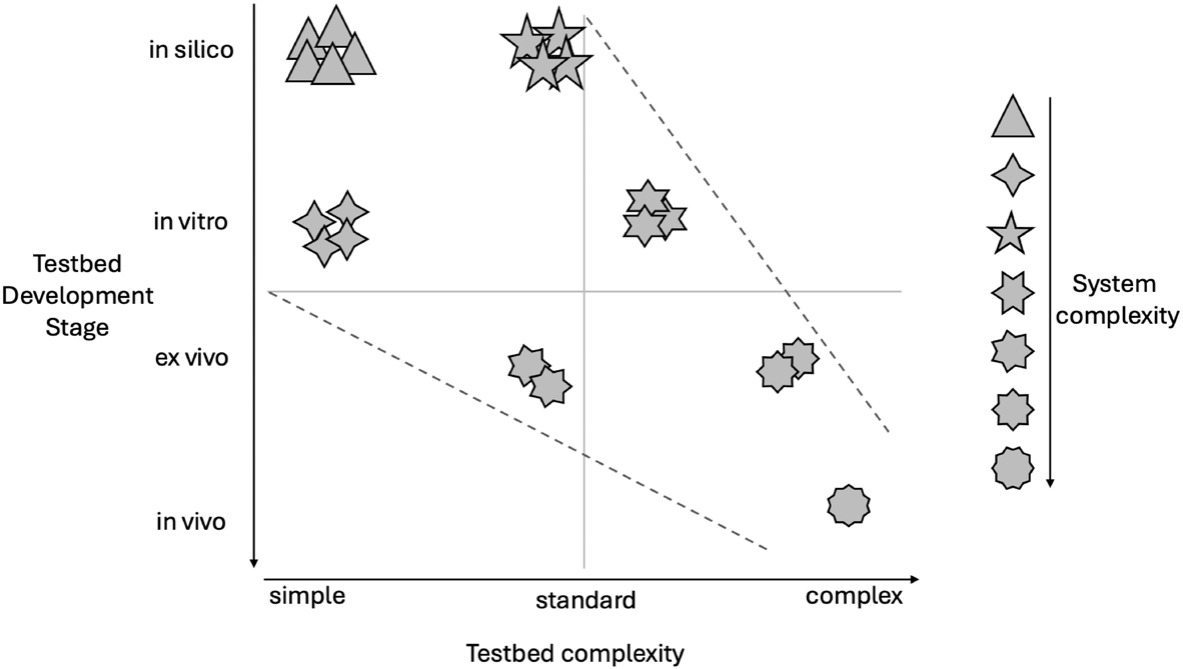
Evolution of testbed complexity with feature complexity. Examples of system complexity, simple in silico, simple in vitro, standard in silico, standard in vitro, standard ex vivo, complex ex vivo, and complex in vivo, are shown in Figures S1 to S7, respectively.

The development process of robotic MT relies on 4 distinct testbed methods: in silico, in vitro, ex vivo, and in vivo, each serving specific evaluation and validation purposes. Although in silico and in vitro testbeds serve as primary platforms for early‐stage research, we acknowledge that implementing experimental factors, such as simulated blood flow and pulsatility, might be ideal as they are recommendations from the Delphi consensus. However, they present considerable challenges, such as increased financial and logistical cost to develop computational or physical experimental testbeds. Introducing such complex elements at the outset could potentially impede innovation in this emerging field^37^; we therefore recommend permissiveness at the outset with a “minimum viable product”; if the engineering capability and budget are not constrained, these physiological factors can be introduced at the outset. In this position statement, we address these challenges by proposing a progressive approach that emphasizes accessibility for researchers during initial experimental stages while systematically incorporating additional features recommended from the Delphi consensus to enhance realism and allow more comprehensive experimental analyses. Early development of robotic MT systems, whether AI assisted or not, focuses on evaluating specific features using simplified testbeds. As development progresses toward regulatory certification for clinical use, the complexity must gradually increase to match real interventional scenarios. Throughout the development cycle of an MT robotic system, testbeds transition from in silico to in vivo, with mandated complexity progressing from simple to complex configurations, as illustrated in Figure 5. It is important to recognize that each development stage has inherent complexity limitations. For example, experiments conducted in silico are unlikely to achieve full equivalence with those conducted in vivo, whereas in vivo testing cannot be simplified to control variables below certain inherent complexity levels.

This approach creates an innovation funnel, which facilitates the rapid evaluation of multiple concepts at the beginning while ensuring the rigorous testing of the MT robotic systems at later stages. We define 3 different levels of complexity across the modality spectrum of in silico, in vitro, ex vivo, and in vivo: simple, standard, and complex:

- The simple level incorporates realistic vessel anatomies compatible with guidewire and catheter use.
- The standard level builds on this foundation and introduces deformable vessels.
- The complex level achieves maximum realism by incorporating blood flow, pulsatility, and disease conditions (such as atheromatous plaques).

A typical evaluation process begins with accessible methods, including simple in silico and in vitro testbeds, and advances to more complex ex vivo, and ultimately in vivo experiments. To promote research advancement, maintaining a low entry barrier for comparable and reproducible evaluation is crucial. Open‐source simulation frameworks and benchmarks, such as stEVE (simulated EndoVascular Environment^38^), provide simple in silico testbeds that enable reproducible evaluation and globally comparable results.

These approaches can be further validated using simple in vitro testbeds, featuring rigid transparent vessel phantoms with overhead camera imaging feedback, eliminating the absolute requirement for fluoroscopy systems. Commercial simulators, like the Cathis (CATHI GmbH, Germany) or the VIST System (Mentice AB, Sweden) enable in silico testing at standard and early complex levels. AI could also be used to generate anatomically complex in silico testbeds, such as synthetic vascular models derived from 3‐dimensional spline‐based methods for training and validation of autonomous systems.^39^ Similarly, ex vivo testing can achieve standard and complex levels by incorporating flexible vessel systems and pulsatile pumps. Many essential components are commercially available, including sophisticated vessel systems from Elastrat Sarl (Geneva, Switzerland) or TrandoMed (Ningbo Trando 3D Medical Technology Co, Ltd, Ningbo, China), and pulsatile pumps for realistic blood flow from ViVitro Labs, Inc (Victoria, Canada) or BDC Laboratories (Wheat Ridge, Colorado, USA). A diverse array of devices should also be used during in vitro testing to reflect those available for use in clinical practice.

Ex vivo evaluation can achieve standard complexity by substituting the in vitro testbed's vessel system with porcine or human organs (including placenta), although our focus in this position statement is on complex ex vivo testbeds, such as human cadavers, which enhance realism. In vivo studies, typically conducted in living porcine models, inherently represent complex testbeds. Although ex vivo human cadaver studies offer superior anatomic accuracy compared with in vivo porcine studies, certification regulations, such as US Food and Drug Administration or Medical Device Regulation of the European Union requirements, must guide the pathway toward clinical studies.

This innovation funnel method enables the development and evaluation of numerous potential candidates while progressively enhancing clinical validity at each stage. Examples of system complexity, simple in silico, simple in vitro, standard in silico, standard in vitro, standard ex vivo, complex ex vivo, and complex in vivo, are shown in Figures S1 to S7,^40^, ^41^ respectively.

### Effectiveness Metrics

We identified 2 macroclasses of effectiveness metrics. One for in silico, in vitro, and ex vivo development stages, which focus on technical assessment and are related to navigation (eg, path following error). For the in vivo development stage, there is a second macroclass of metrics that is more clinically oriented (eg, mTICI). The Delphi consensus recommendations also show that using a range of metrics would be ideal for measuring effectiveness. However, as with testbed recommendations, we acknowledge that some of these metrics may be challenging to implement and, therefore, we advocate a pragmatic approach in this position statement.

The main practical challenge relates to measuring forces even though several methods for their assessment exist. Measuring contact forces in silico during navigation is feasible with the inherent advantage that measurement does not mechanically interact with the testbed or device.^33^, ^42^, ^43^ Measuring contact forces on the vessel walls has also been successful in vitro, by either using a force sensor underneath the phantom base^44^, ^45^ or equipping phantoms with surface sensors.^46^ Base or tip forces can also be measured by either equipping the endovascular device with sensors^47^ or extending the robot drivetrain.^48^ However, recording such forces may require additional equipment, which could compromise MT performance. Moreover, force measurements may only be considered a useful metric if a consensus existed on what should be considered excessive. With no meaningful way of measuring applied forces in patients and relating these to clinical safety, such a consensus threshold may, for now, remain elusive. Instead of using predefined thresholds, it may be more meaningful to use expert‐recorded force profiles as a reference. The difference between the force applied by the robotic system and that of an expert demonstrator, or a similar derived metric, could serve as a measure of effectiveness. However, no suitable method for this has been established in current research.

Similarly, consideration must be given to “path following error” (in silico and in vitro). Current metrics utilize the centerline of the vessel to determine the similarity of navigation path taken. However, this may not be ideal as the vessel centerline is not necessarily the best path to take during MT navigation. Instead, a more representative measure may be to prerecord the navigation of experts, and use the difference in path against the closest expert as an effectiveness metric.

We propose that early research with a low TRL should focus on key effectiveness metrics of success rate, number of phases or steps, number of failures, number of handling errors made, path length, and procedure time, which can be measured effectively across all testbed types. As the TRL of the work increases, metrics such as “fluoroscopy time” and “instrument tip speed” should be used to provide a more comprehensive analysis. For metrics such as path following error, and contact forces at the instrument base, instrument tip, and vessel walls, more research is required to understand if and how these metrics impact navigation behavior and potential clinical outcomes during AI‐assisted MT, in particular.

### Clinical Perspective

Until now, there has been no distinct pathway laid out for developing robotic MT in a way that will clearly demonstrate safety, clinical efficacy, and utility (including cost‐effectiveness), all of which need to be demonstrated to introduce a new technology into routine clinical care.

Although AI‐assisted or autonomous robotic MT has limited direct clinical relevance at present, the pace of development is extremely rapid, so we must look ahead to what may be the relatively near future of MT practice and ensure any robotic system and/or AI assistance can show safety, efficacy, and utility against agreed standardized criteria. However, patient safety is critical and must override other considerations as regards clinical practice.

This position statement and integral Delphi process have developed clear consensus for several components in the development, introduction, and assessment of robotic MT. Consensus offers innovators, industry, researchers, and clinicians an agreed appropriate pathway to achieve safe but rapid development, assessment, and ultimately implementation of AI‐assisted robotic MT into routine clinical practice. The effectiveness measures agreed on for efficacy and safety in vivo are well understood, validated, and widely used in MT research.^4^, ^7^

It will be imperative going forward that the safety metrics recommended in this position statement (eg, contact forces of vessel walls and instruments) are developed and independently validated. As an early priority next step, it would be vital to correlate in detail how in vitro measurements (such as contact forces) relate to in vivo safety and complications (eg, risk of perforation, intracranial hemorrhage, dissection, and distal embolization). This will require close collaboration and partnership between industry, academia, and neurointerventional clinicians.

### Patient Perspective

The patient representatives involved in this initiative have each experienced the devastating impact that a stroke can have on families. When a stroke is diagnosed, the urgency for intervention is unquestioned. However, the procedures are complex and risky for the patient, requiring specialized interventions. Robotic MT (with or without AI) has the potential to dramatically change this.

We see that this position statement is the first step in addressing an uncertainty that is delaying progress toward the introduction of robotic MT (with or without AI). One reason for this delay is that there is no evidence or data comparing manual MT and robotic MT (with or without AI) and, therefore, it is unknown whether there is an advantage of one approach over another. Clinical participants were united in the view that common reference standards do not exist, nor do standard measures of effectiveness of treatments, but that their development was necessary.

From the standpoint of patient representatives, there appears to be extensive agreement from the professions on why it is important that robotic MT (with or without AI) is translated. There also appears to be overall agreement on what is important in terms of which “biological” characteristics need to be included (such as atheromatous plaques), and which robotic behaviors are important.

Anyone with experience of this condition will want this vision to come to fruition in the clinic as quickly as possible. However, it is understood that this new technology will require rigorous proving through evidence, including clinical trials, to move beyond TRL 3. Clearly, the next steps must capitalize heavily on the consensus to date; and patients would then expect multiple studies, but, crucially, based on this position statement derived from the Delphi process. In addition, but for future consideration, the patient community will have concerns over the evolution of robotic MT procedure toward a fully autonomous one using AI. The role of oversight, and what back up is considered acceptable in remote locations should complications occur, the development of trust in AI, and how consent would be given for this procedure will be suitable future topics for the START initiative to address.

### Health Economics

Health technology assessment with health economic evaluation can be performed at any time during the development of a new technology and ideally should take an iterative approach alongside the development process.^49^ At this early stage in the development of robotic MT (with or without AI), a developer‐focused health technology assessment approach is recommended.^50^ This approach is essential for clearly identifying the clinical need and for generating robust evidence to support it.

Importantly, the developer‐focused health technology assessment should be undertaken even before any AI‐assisted robot has been finalized so that the developers can respond to the findings, such as changing the overall design, use case, target population, and/or place in the clinical pathway. The process of following a developer‐focused health technology assessment approach will allow the technology developers to build evidence generation plans convergent on their technology development pathway. Qualitative research methods should be used to capture content from semistructured discussions with regulators, policy decision makers, and expert opinion. For health economic evaluation to be useful at this stage in the development pathway, the focus of this work must be on reducing any uncertainty in the health economic model while robotic MT (with or without AI) is undergoing testing. This can be done by exploring studies of similar techniques if they exist (eg, summarizing endovascular robots with AI assistance^17^), or previous generations of the technology (eg, showing neuroendovascular robots without AI assistance^25^) as well as exploring the reliability of the experts' opinions on plausible ranges of effect sizes,^51^ gathering detailed information on who will be using the robot, and the impact that it will have on workflow within the clinical environment where it will be deployed. Other work could be to use a Markov model to simulate health outcomes and costs of the treatment over a lifetime time horizon, as has already been performed for MT.^52^ Cost‐based analyses would incorporate patient and social benefits from intervening earlier, including more rapid discharge to home, more rapid return to work, and more long‐term independence. Once metrics have been decided, using this position statement, which has (1) determined which metrics are appropriate and (2) articulated a developer‐driven approach to deciding which of these metrics to use, then simulations can be run to understand the sensitivity of robotic MT (with or without AI), to each of these parameters, and to prioritize the collection of de novo data where there is large uncertainty in the values.

At this stage of the technology, it is recommended that developers focus on articulating and quantifying a value proposition by using qualitative research methods to capture users' and patients' lived experiences as well as some simple budget impact analyses on existing health care services. There will be some commonality between different robots able to perform MT (with or without AI), and in future work, START could enable capture of baseline data.

### Ethical Consideration for Implementation

Autonomous or assisted navigation will be used to mitigate the risks of a teleoperation by an expert, and/or to upskill local generalist operators at the patient's local site. To optimize accuracy, AI‐assistive technology will benefit from data‐driven approaches with physical modeling of biology, physiology, and interventional devices, which means health data need to be shared, and sharing should occur within an ethical framework.

Patient safety and informed consent, also highlighted within the Clinical and Patient Perspective Positions above, respectively, must be given top priority in the ethical implementation of new technologies, guaranteeing that patients are completely aware of the risks and benefits. Patient's opinions should continue to be addressed with engagement potentially through surveys or representation during stakeholder meetings. It is noteworthy that START accrued experience of “patient and public involvement” before the initial incubator day. Here, stakeholders were concerned that vessels would be damaged by guidewires because of a physical reality gap caused by robotic teleoperation. The concept of AI assistance was, therefore, considered an important mitigation strategy for increasing safety during teleoperation.

Ongoing observation after implementation of AI‐assisted robotic MT is also necessary to monitor and resolve any unforeseen implications, such as differences in outcomes or access across various patient groups, especially those in the most rural regions, where back up is limited should complications occur. After implementation, there is also an ethical imperative to consider technology sustainability. Central to this is standardized billing and coding procedures that appropriately account for the intricacy of these technologies and will ensure their long‐term viability and fair compensation for all medical professionals involved.

To ensure ethical and regulatory compliance with health care standards, and to streamline the respective development process, our endeavor should also concentrate on establishing regulatory pathways that enable translation of AI‐assisted robotic MT from early developmental stages to clinical practice. Here, regulatory bodies need to be involved early on (these are nation specific, and it is beyond our remit to detail these; but in the United States, for example, this would include those bodies involved in state‐based licensure to streamline dual licensing through the Interstate Medical Licensure Compact, or cross‐state hospital credentialing, or even policy reform for telehealth reciprocity and federal licensure). Before full implementation of robotic AI‐assisted MT, consideration should also be given to cybersecurity and the transmission of information required to enable teleoperated robotics, ensuring that all data remain secure in critical interventions.

We can improve the integration of cutting‐edge technologies and procedures in clinical settings by taking a comprehensive approach to these challenges, which will eventually improve access to patient care and endovascular intervention outcomes globally. We suggest convening a dedicated future incubator day and creating a follow‐up position statement to address the broad implementation, and related ethical issues.

### Strengths and Limitations

The mixed group of stakeholders from 5 countries and 2 continents, and the 100% yield for every step in the Delphi process, suggests that the results represent a reasonable estimate of best practice for optimizing testbeds and effectiveness metrics. Our item list for the Delphi process was comprehensive, could be updated during the process, and was likely to capture the key endovascular testbed items and the key effectiveness metrics to be considered important by the panel. Nonetheless, this study has limitations. The panel was small, and unlikely to be representative of all stakeholders. For example, despite START being announced at multiple international conferences and meetings, many experts may be unaware of the initiative or may not have had time to contribute. Moreover, although the Delphi process was designed with care, responses may reflect varying interpretation of the questions. Furthermore, the deliberately narrow focus on testbeds and effectiveness metrics was at the detriment of focusing on other important aspects of AI‐assisted robotic MT. However, the START initiative has only just begun, and as shown above, there are already recommendations for future developments, including new position statements. Other avenues that would benefit the community through consensus also include procedural training. Additional position statements, especially if published within 12 to 24 months of this one, will together lead to greater translational impact.

## CONCLUSIONS

Currently, AI‐assisted robotic MT has limited clinical relevance. However, the pace of development of robotics and AI is so rapid that transformative solutions are likely to be imminent.^53^ Thereafter, the technology can be taken beyond TRL 3 and implemented once key translational milestones are passed (Table S4). Therefore, we must ensure that any robotic system and/or AI assistance can show safety, efficacy, and utility against agreed standardized criteria. Through a Delphi consensus, this position statement has established the first step in this process by establishing recommendations for testbeds and effectiveness metrics to develop and validate AI‐assistive robotic MT. We have identified a practical approach to judiciously select these established recommendations on a case‐by‐case basis, recognizing the differing requirements for testbed realism, and therefore complexity, during AI‐assistive robotic MT at different developmental stages. We endorse a similar practical approach for effectiveness metrics. We have also identified patient safety to be central, and yet not perceived to be a risk, to the development of this technology. One requisite patient safety task needed now is to correlate in vitro measurements (eg, contact forces) to in vivo complications (eg, risk of perforation, intracranial hemorrhage, dissection, and distal embolization). Health economic evaluation can also begin now, even at a low TRL. A follow‐up position statement will address implementation strategies and ethical considerations.

## Appendix

### Other Members of START

Frédéric Clarençon (Department of Interventional Neuroradiology, Sorbonne Université, APHP, Pitié‐Salpêtrière Hospital, Paris, France), Adnan Siddiqui (Department of Neurosurgery, State University of New York at Buffalo, Buffalo, NY), David Bell (Remedy Robotics, San Francisco, CA), Nikola Fischer (School of Biomedical Engineering and Imaging Sciences, Kings College London, London, UK), Kawal Rhodes (School of Biomedical Engineering and Imaging Sciences, Kings College London, London, UK), Christos Bergeles (School of Biomedical Engineering and Imaging Sciences, Kings College London, London, UK).

### Society Endorsements

Society of Vascular and Interventional Neurology (United States) and United Kingdom Neurointerventional Group.

## Sources of Funding

This work was supported by the MRC IAA 2021 Kings College London (MR/X502923/1) and the Wellcome EPSRC Centre for Medical Engineering at King's College London (203 148/Z/16/Z). AH is supported by grants from: Asahi, Balt, Scientia, Valley Baptist, GE Healthcare, and Viz.ai. MO would like to thank the FOD Economy in the call of 5G Pilot‐Projects (MSCAA) for funding this work, and the internal KU Leuven C3 project (RoboGuide).

## Disclosures

AH is a consultant/speaker at: Medtronic, Microvention, Stryker, Penumbra, Cerenovus, Genentech, GE Healthcare, Scientia, Balt, Viz.ai, Insera Therapeutics, Proximie, NeuroVasc, NovaSignal, Vesalio, Rapid Medical, Imperative Care, Galaxy Therapeutics, Route 92, Perfuze, CorTech, Shockwave, Toro, and Xcath. AH is a Principal Investigator for: COMPLETE study—Penumbra, LVO SYNCHRONISE—Viz.ai, MARRS—Perfuze, RESCUE‐ICAD—Medtronic. AH is on the Steering Committee/Publication committee member for: SELECT, DAWN, SELECT 2, EXPEDITE II, EMBOLISE, CLEAR, ENVI, DELPHI, DISTALS, and Rapid Pulse. AH is a DSMB for the COMAND trial. DM consults for a variety of medical/surgical robotics companies, and is the original creator of the Mentice VIST simulator. FMU is cofounder and shareholder of Ophthorobotics AG, whose interests are unrelated to the presented work. MP, RF, and MK are employed by Siemens Healthineers. RC is a consultant/speaker at: Terumo Neuro, Stryker, J&J Neuro, Accandis, Phenox, and Penumbra. RC has a collaboration (noncommercial) with Mentice AB. RF is cofounder and shareholder of Tibaray Inc, whose interests are unrelated to the presented work. TV is cofounder and shareholder of Hypervision Surgical Ltd, whose interests are unrelated to the presented work. TB has performed Consultancy and Speakers Bureau speakers services for Siemens Healthineers, Medtronic, Bayer, and Core laboratory for Microvention.

## Supporting information

Tables S1–S4
Figures S1–S7
References 40–41
