# Supplementary material for: A Position Statement on Endovascular Models and Effectiveness Metrics for Mechanical Thrombectomy Navigation, on Behalf of the Stakeholder Taskforce for Artificial Intelligence–Assisted Robotic Thrombectomy (START)
Source: J Am Heart Assoc. 2026 Mar 28;15(7):e044931. doi: 10.1161/JAHA.125.044931 (PMC13279122; doi:10.1161/JAHA.125.044931)
Supplement: Supplementary file 1 — Tables S1–S4 Figures S1–S7 References 40–41 [file JAH3-15-e044931-s001.pdf]

SUPPLEMENTAL MATERIAL

Table S1. Benefits and risks without consensus after three rounds for robotic MT, both with and without AI (across all developmental stages of robotic MT from *in silico* to *in vitro*). Percentage agreement for each benefit and risk was taken after all rounds.

Domain	Benefit/Risk	Agreement
**Benefit**	To decrease patient radiation exposure	68% (15/22)
	To decrease patient contrast exposure	41% (9/22)
	To identify the best devices or approach for a particular patient	68% (15/22)
	To support training of new operators	68% (15/22)
	To standardize procedures	64% (14/22)
	To increase instrument dexterity and improve intuitive navigation (reducing the workload of operator)	73% (16/22)
**Risk**	Ethical issues	45% (10/22)
	Patient safety	64% (14/22)
	Lack of pre-clinical or *ex vivo* ways to test the equipment	68% (15/22)
	Societal concerns towards robots and/or AI	36% (8/22)
	Patient reluctance to undergo robotic treatment	14% (3/22)
	Adoption by a neurointerventional radiologist of the technology	50% (11/22)

Table S2. Non-consensual items after exploring which experimental factors were considered important for each developmental stage testbed (both with and without AI). Percentage agreement for each item was taken after all three rounds.

Developmental stage	Factor	Very Effective/Somewhat Effective
in silico	Simulated respiration	43% (9/21)
in vitro	Simulated respiration	35% (7/20)
***ex vivo* (human cadaver)**	Simulated pulsatility	55% (11/20)
	Simulated blood flow	20% (4/20)

Table S3. Non-consensual items after exploring which effectiveness measures were important for each developmental stage (both with and without AI) as well as during *in vivo* clinical assessment. Percentage agreement for each item was taken after all three rounds.

Developmental stage	Effectiveness Measure	Very Effective/Somewhat Effective
in silico	Path length	76% (16/21)
	Instrument tip speed	76% (16/21)
	Instrument tip acceleration	67% (14/21)
	Volume of contrast agent	48% (10/21)
	Number of guidewire tip touches on the vessel wall)	75% (15/20)
in vitro	Instrument tip acceleration	75% (15/20)
	Volume of contrast agent	55% (11/20)
	Fluoroscopy time	75% (15/20)
	Number of guidewire tip touches on the vessel wall)	75% (15/20)
ex vivo	Path length	71% (15/21)
	Path following error	70% (14/20)
	Instrument tip speed	65% (13/20)
	Instrument tip acceleration	55% (11/20)
	Volume of contrast agent	55% (11/20)
	Number of guidewire tip touches on the vessel wall)	70% (14/20)

Table S4. An example of proposed technical, clinical, economic and regulatory milestones for safe translation.

Category	Key Performance Indicators (KPIs) / Milestones
**Reproducible technical KPIs**	1. Device compatibility: Demonstrated use with standard devices (guide/sheath, aspiration, stent-retrievers) across multiple vendors; rapid tool-change workflow.
	2. Operating room integration and interoperability: Compatible with biplane angiography suites; adequate cyber-security (e.g., ISO 27001/NIST), medical IT-network risk management (IEC 80001-1).
	3. Teleoperation performance: Adequate end-to-end latency, jitter, link availability; automatic fail-safe modes and "safe stop/withdraw" on dropouts.
**Prospective human data**	1. Showing non-inferior morbidity and mortality outcomes.
	2. Faster patient access.
**Financial viability**	1. Confirmed commissioned network pilots: funding/reimbursement including training of staff.
	2. Health-economic analysis: cost-effectiveness (Incremental Cost-Effectiveness Ratio (ICER), Quality-Adjusted Life Years (QALYs)), capacity gain (cases/week), avoided transfers; demonstrate feasibility of different time models including 24/7 coverage.
**Financial viability**	1. US: FDA Investigational Device Exemption (IDE) study and Premarket Approval (PMA).
	2. EU/UK: UK Conformity Assessed (UKCA)/Conformité Européenne (CE) marking under EU MDR/UK MDR.
	3. Expanding state-based and/or country-based licensure.

Figure S1. Example of simple *in silico* testbed.

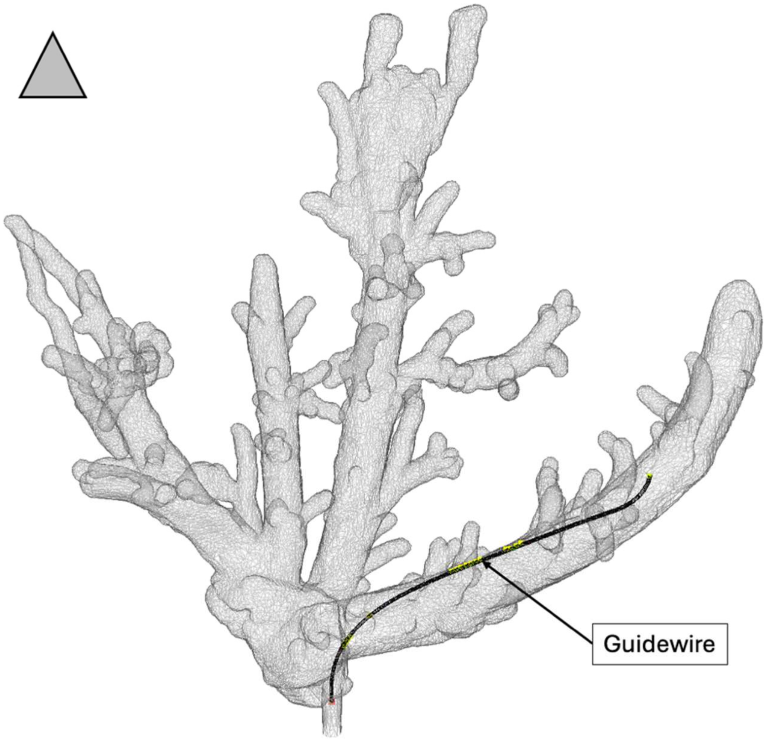

Figure S2. Example of simple *in vitro* testbed.

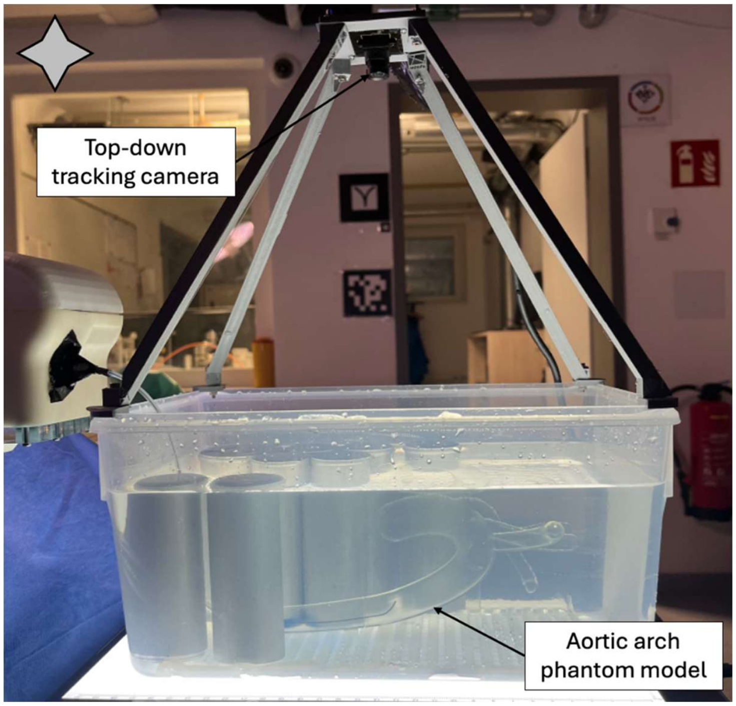

Figure S3. Example of standard *in silico* testbed.

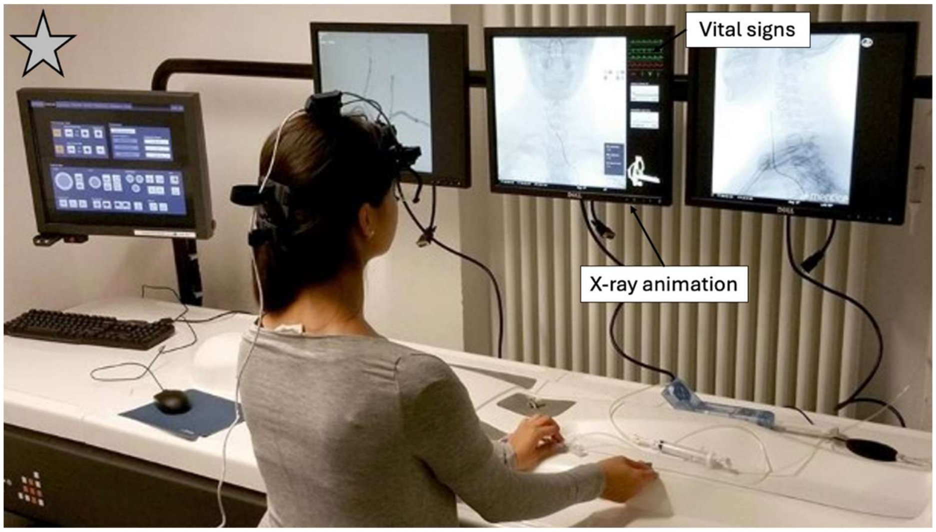

Adapted from: Kreiser et al. 2021 [52]. Reused under the terms of the Creative Commons Attribution 4.0 International License (CC BY 4.0; http://creativecommons.org/licenses/by/4.0/). The image has been cropped and annotated by the authors.

Figure S4. Example of standard *in vitro* testbed.

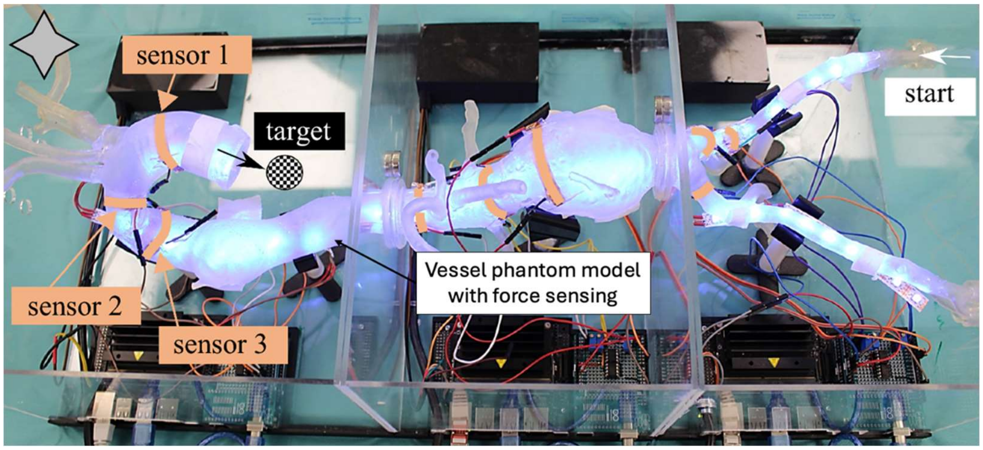

Adapted from Fischer et al. 2023 [44]. Reused under the terms of the Creative Commons Attribution 4.0 International License (CC BY 4.0; http://creativecommons.org/licenses/by/4.0/). The image has been cropped and annotated by the authors.

Figure S5. Example of standard *ex vivo* testbed.

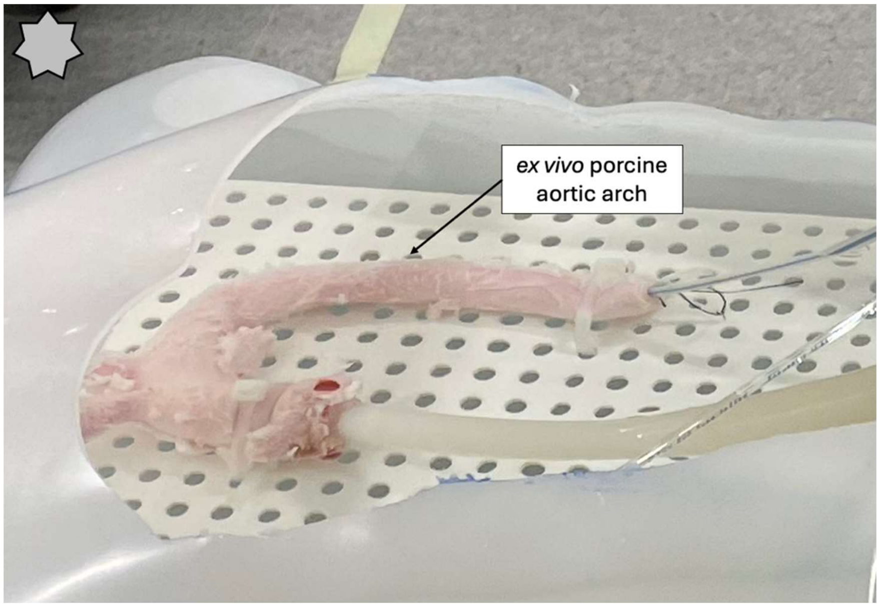

Figure S6. Example of complex *ex vivo* testbed.

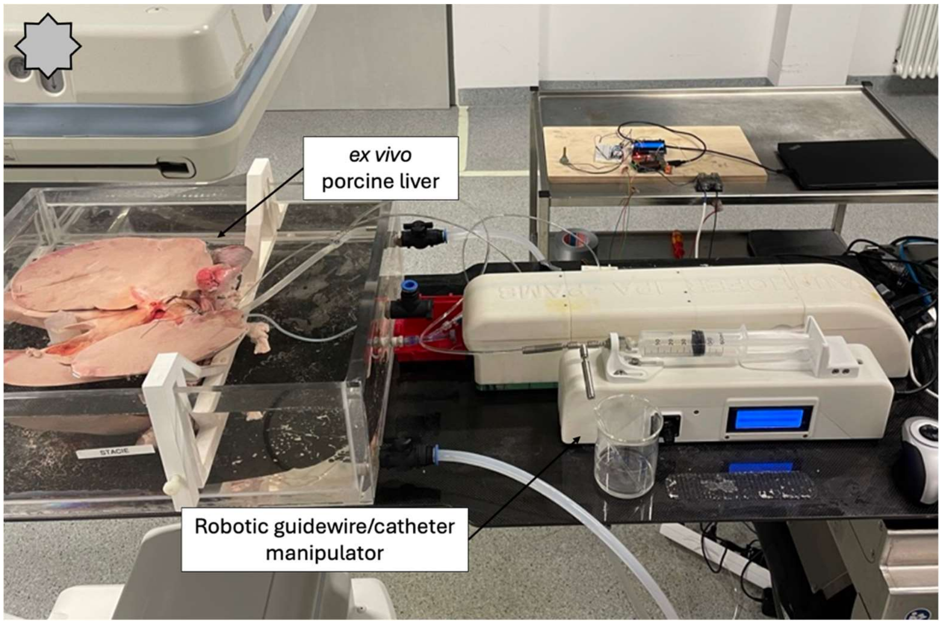

Figure S7. Example of complex *in vivo* testbed (porcine model).

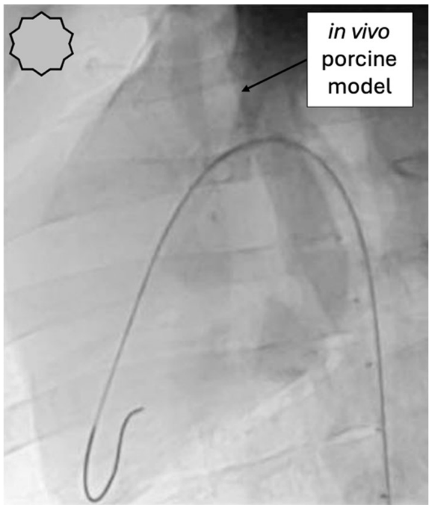

Adapted from Peng et al. 2023 [53]. Reused under the terms of the Creative Commons Attribution 4.0 International License (CC BY 4.0; http://creativecommons.org/licenses/by/4.0/). The image has been cropped and annotated by the authors.
